# 2D amorphous solids for sub-nanometer scale devices

**DOI:** 10.1186/s40580-024-00453-2

**Published:** 2024-11-24

**Authors:** Hyeonseo Jang, Hyeonju Kim, Gayoon Kim, Suyeon Cho, Heejun Yang

**Affiliations:** 1 Division of Chemical Engineering and Materials Science, Graduate Program in System Health Science and Engineering, Ewha Womans University, Seoul, 03760 Korea; 2 Department of Physics, Korea Advanced Institute of Science and Technology (KAIST), Daejeon, 34141 Korea

**Keywords:** 2D amorphous solids, Phase transition, Sub-nanometer scale devices

## Abstract

Amorphous solids are a type of condensed matter characterized by the absence of long-range order in their lattice structure. However, they still exhibit short- or medium-range order, which contributes to their versatile local and global electronic and chemical properties. Recently, 2D amorphous solids have gained attention for their exceptional mechanical and electronic features, which are unattainable in conventional crystalline materials. This review highlights the physical properties of ultrathin 2D amorphous solids, which are formed through covalent bonding and feature polyhedron structures with shared edges and corners. Two notable examples of 2D amorphous solids include honeycomb-structured nanosheets with mixed hybrid orbitals and layered materials with reduced coordination numbers of the elements. We provide an in-depth discussion of (1) the phase transition between crystalline and amorphous phases in 2D solids, (2) advanced synthetic methods for producing high-quality amorphous films with precise thickness control, and (3) the potential applications of sub-nanometer scale 2D amorphous solids. Lastly, we explore their potential to revolutionize the design of highly versatile electronic devices at sub-nanometer scales.

**Graphical Abstract:**

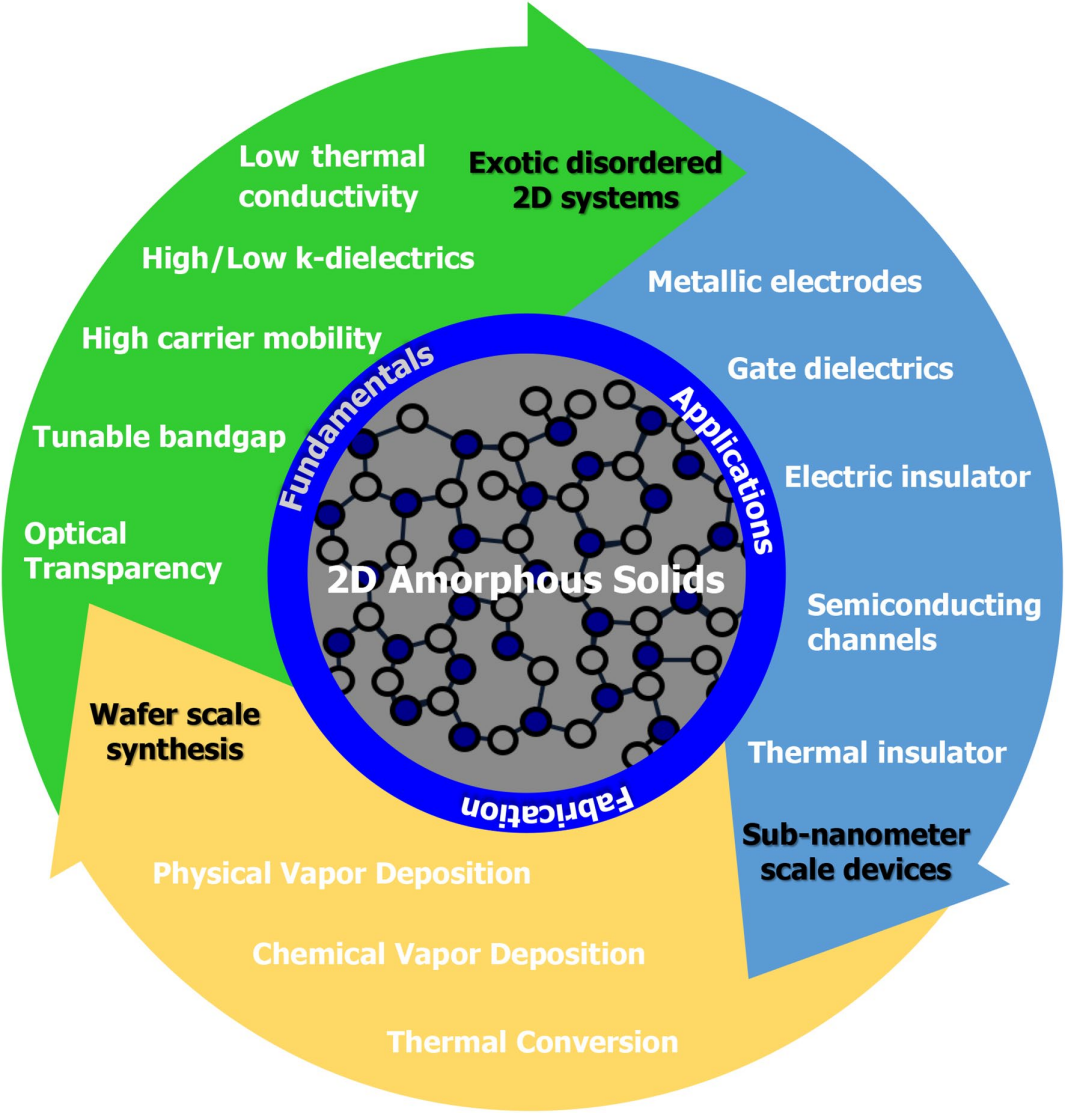

## Introduction

The tremendous success of the semiconductor industry over recent decades has driven rapid advancement in scaling down electronic devices. Simultaneously, we are entering a new era of breakthroughs in artificial intelligence, largely powered by neuromorphic devices. Both trends underscore the growing need for novel materials to enable atomic-scale device operation and reduce energy consumption [1–4]. While sub-1 nm-scale science and technology are reportedly poised to shape the future of the semiconducting industry, the primary bottleneck remains the development of suitable materials. This challenge calls for a new research approach to meet the demands of next-generation electronic devices.

Amorphous solids are a class of materials where the long range of periodicity (longer than 20 Å) is not present or valid [5, 6]. Therefore, amorphous solids cannot be explained by traditional theories of solid-state physics, which rely on the assumption of infinite (periodic) lattices with high symmetry. Many interesting amorphous solids such as glasses, plastics, and polymers have been reported so far in the research fields of electronic devices, catalysts, solar cells, and biomaterials [7–10]. The unique physical and chemical properties of amorphous solids require reexamination through more adaptable and realistic approaches.

Recent studies have reported significant breakthroughs with various amorphous solids, particularly atomically thin, two-dimensional (2D) systems, such as carbon [11, 12], boron nitride (BN) [13], and transition metal dichalcogenides (TMDs) [14, 15]. For instance, amorphous BN has been synthesized and exhibits an exceptionally low dielectric constant [13], while another study found that amorphous silicon nitride (SiN) exhibits a high dielectric constant [16]. These findings highlight the diverse and tunable properties of amorphous solids, which make them promising candidates for advanced devices.

Intriguing electrical/thermal conductivities, as well as topological states, have been discovered in various amorphous solids [17, 18]. Despite their structural disorders, certain amorphous solids demonstrate remarkable mechanical stability, making them suitable as media for ion and carrier transport [19, 20]. These exceptional physical and chemical properties could pave the way for future novel electronic (e.g., neuromorphic devices) and energy-harvesting devices [21–27].

**Fig. 1:**
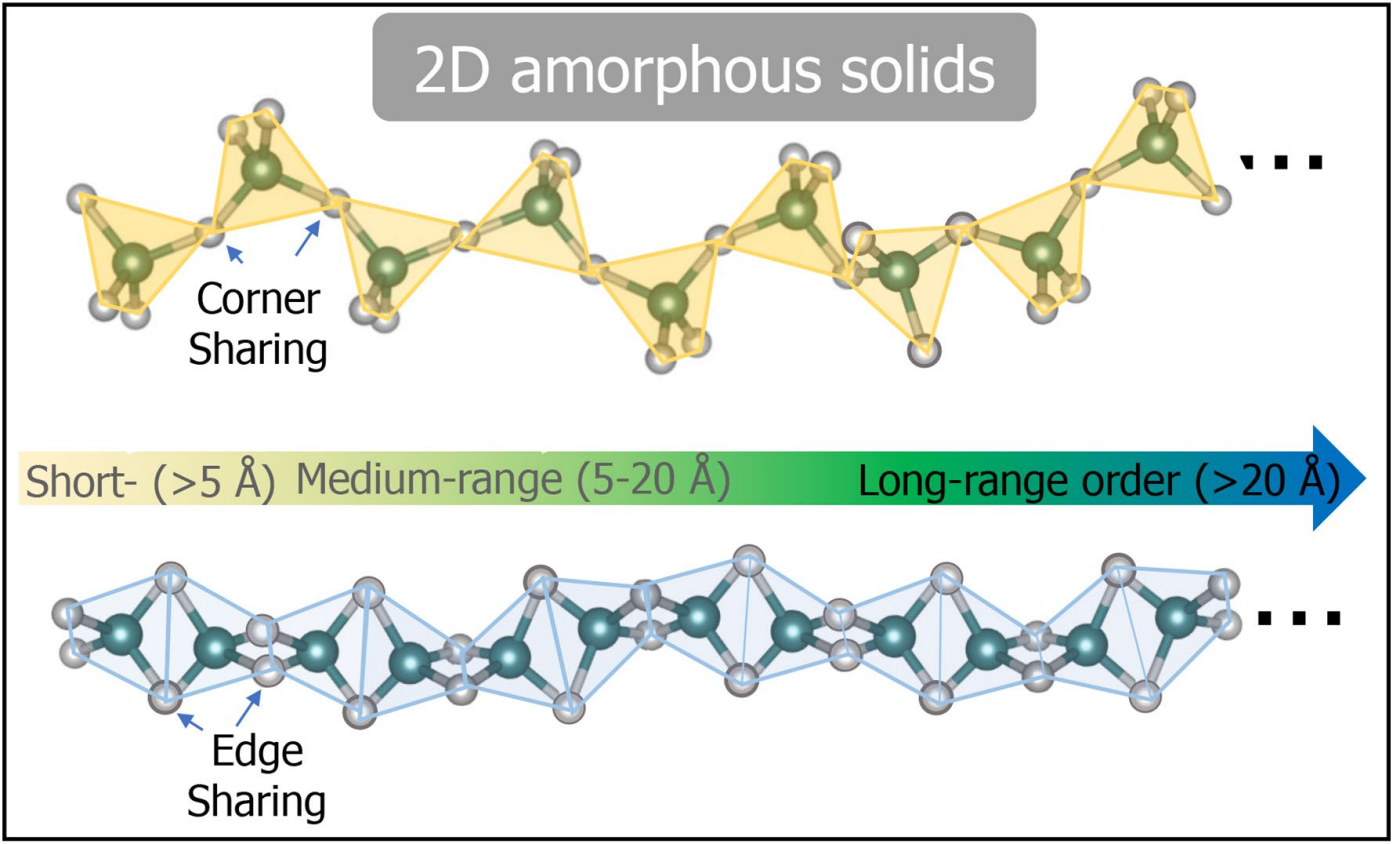
2D amorphous solids are made by sharing the edges or corners of polyhedrons

To develop a new class of materials functional at a sub-1 nm scale, some researchers have turned to 2D amorphous solids, which exhibit a medium-range order (5–20 Å) rather than a short-range order (2–5 Å) (see Fig. 1). While many amorphous solids have been reported with short-range ordering, medium-range or even longer-length scale order in amorphous solids has remained elusive [5, 28]. Yang et al.. reported that some amorphous metallic alloys exhibit both short-range and medium-range order at the single-atom level, leading to crystal-like superclusters with translational order [29]. According to Elliott et al..‘s classification, amorphous solids with medium-range order can form specific geometries, such as 0D nanodots, 1D nanowires, and 2D nanosheets, by sharing the edges or corners of polyhedrons. These structures offer strong mechanical strength, which is crucial for the advancement of next-generation materials [6, 30].

This review narrows its focus to sheet-like, 2D amorphous solids with thicknesses down to the sub-nanometer scale. Given the structural instability of materials at this scale, Chap. 2 will first delve into the fundamental properties of amorphous solids. Chapter 3 will explore both synthetic and post-synthetic strategies for fabricating amorphous solids and related patternings, with particular attention to 2D amorphous solids. Finally, Chap. 4 will discuss recent reports on the various applications of amorphous solids, highlighting their potential in advanced device technologies.

## Fundamentals

The initial motivation for extensive research on crystalline-amorphous phase transition was to advance phase change memory (PCM) and neuromorphic devices. For these applications, memory devices must exhibit multiple (at least two) conductance states in their channels. Numerous studies have demonstrated PCM functionality using emerging materials that undergo reversible phase transition between crystalline (conducting) and amorphous (insulating) phases [31–34]. In PCM, the crystalline-amorphous phase transition allows for multiple conductance states by partially or entirely manipulating the materials’ insulating amorphous and conducting crystalline regions [35, 36]. Thus, high-performance PCM devices can be developed with fast switching speed, ultralow energy consumption, and excellent phase stability, utilizing the amorphous phase as an electrically insulating phase. Various binary and ternary chalcogenide films have been employed in PCM devices to facilitate phase transition. This review does not cover materials lacking a 2D geometry, such as GeSbTe, which exhibit Joule-heating-based phase transitions and have been extensively reviewed elsewhere.

In general, amorphous solids are thought of as non-crystalline solids without translational symmetry, preferring short- or medium-range order rather than long-range order. Recently, Illing et al. reported that 2D amorphous solids exhibit the Mermin-Wagner fluctuations, which conserves space homogeneity on the long-range scale [37]. From the thermodynamic point of view, amorphous solids (strictly specified as glassy solids) can be transformed from molten solids by supercooling, where a second-order phase transition at the glass transition temperature undergoes without latent heat [38–40]. It has been reported that melting and freezing in 2D systems progress with a second-order transition via intermediate states, unlike 3D systems with first-order transitions [41–44]. Therefore, research on 2D amorphous solids is interesting and challenging as it allows for fabricating stable and unique structures and studying their fundamental physical and chemical properties.

Mantovan et al. reported that an as-synthesized amorphous binary chalcogenide, composed of 53% tetrahedral and 47% defected-octahedral configurations, could be transformed into a crystalline phase predominantly featuring an octahedral configuration (60%) with minor tetrahedral (> 20%) and defect-octahedral (< 20%) components [45]. In addition, Islam et al. demonstrated a crystalline-amorphous phase transition in an alkali-incorporated ternary chalcogenide, where alkali cations contribute to the formation of a disordered (amorphous) structure under the influence of an applied electric field [46].

**Fig. 2:**
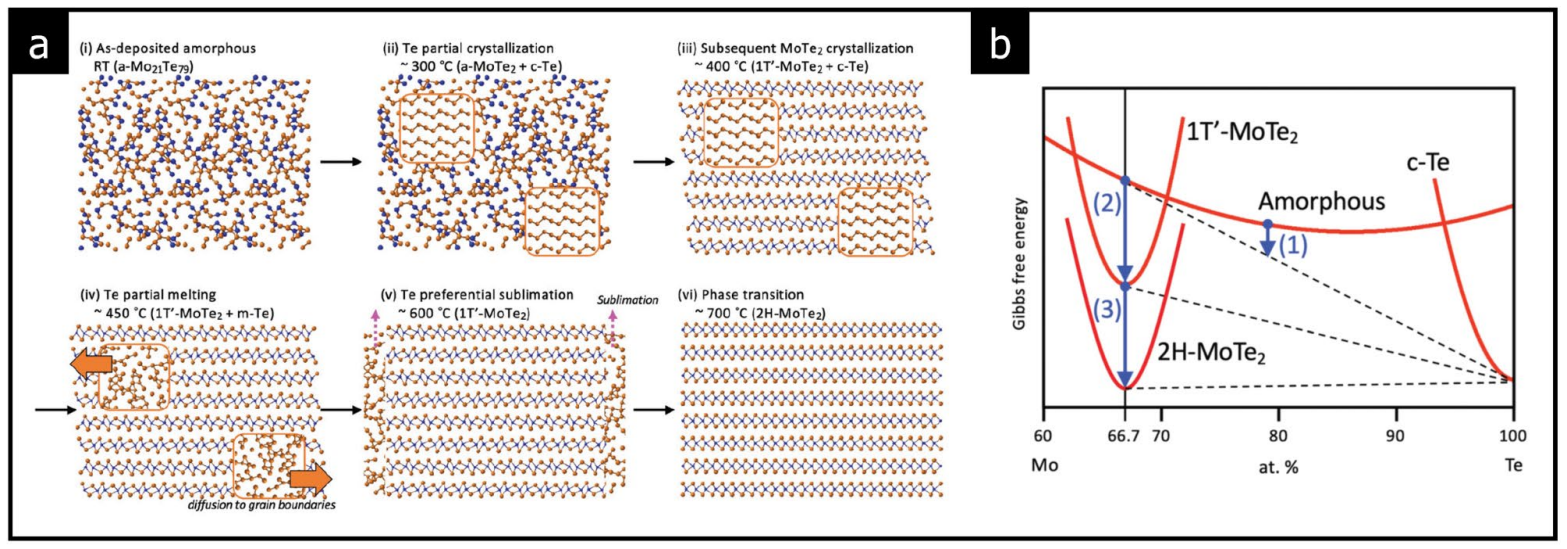
Thermodynamics of the amorphous-to-crystalline phase transition in the Mo-Te system. **a** Te-rich amorphous phase is converted to the crystalline 2H and 1T’ phase in Mo-Te films. **b** Gibbs free energy of the amorphous and crystalline phases in the Mo-Te system. (Reprinted with permission from Ref [14])

A similar crystalline-amorphous phase transition has been more distinctly observed in 2D layered TMDs. In particular, amorphous Mo-Te films exhibit phase transition features with the 1T’, 2 H, and amorphous phases [14, 47, 48]. As illustrated in Fig. 2(a), selective sublimation of tellurium (Te) atoms through heat treatment enables the crystallization of amorphous Mo-Te films into the 1T’ high-temperature phase. With further annealing, the 1T’ phase transitions into a 2 H structure [14]. This transformation can be explained by lattice dynamics governed by the Gibbs free energy of the phases, as shown in Fig. 2(b). Initially, the Te-rich amorphous Mo-Te film undergoes phase separation and transformation along the energy landscape, favoring crystallization. The crystallization is driven by the decreasing Te content, resulting in a transition from an amorphous phase to a more ordered crystalline structure.

As temperature increases and appropriate thermal treatment is applied, the system tends to adopt a lattice structure with lower Gibbs free energy, stabilizing in the 2 H phase. Consequently, amorphous chalcogenides with excess chalcogen elements can transition more readily into crystalline phases than those with exact stoichiometric ratios. Following this principle, Krbal et al. demonstrated that S-rich amorphous MoS_4_, with a locally defined coordination number of 4 (tetragonal), can be transformed into crystalline MoS_2_ with a coordination number of 6 (octahedral). This transition can be attributed to the lower coordination in the amorphous state, which facilitates the incorporation of chalcogen atoms for crystallization [48].

It has been established that the reversible crystalline-amorphous phase transition in thick binary or ternary chalcogenides typically involves a diffusion-induced, successive local phase transition. The diffusion-limited structure of atomically thin 2D materials presents more significant challenges for achieving reversible crystalline-amorphous phase transition compared to 3D bulk materials.

Additionally, the coordination numbers in layered chalcogenides often vary with the thickness of the materials. This complex relationship between stoichiometric ratios and structural stability allows for unique control over their phase through thickness-dependent coordination numbers, enabling the exploration of low-dimensional geometries down to the sub-nanometer scale [49].

As a result, reversible crystalline-amorphous phase transitions have been successfully achieved in a few atomic layers of TMDs. A prominent example is the monolayer of MoS_2_, which exhibits a reversible phase transition between crystalline and amorphous phases [50].

**Fig. 3:**
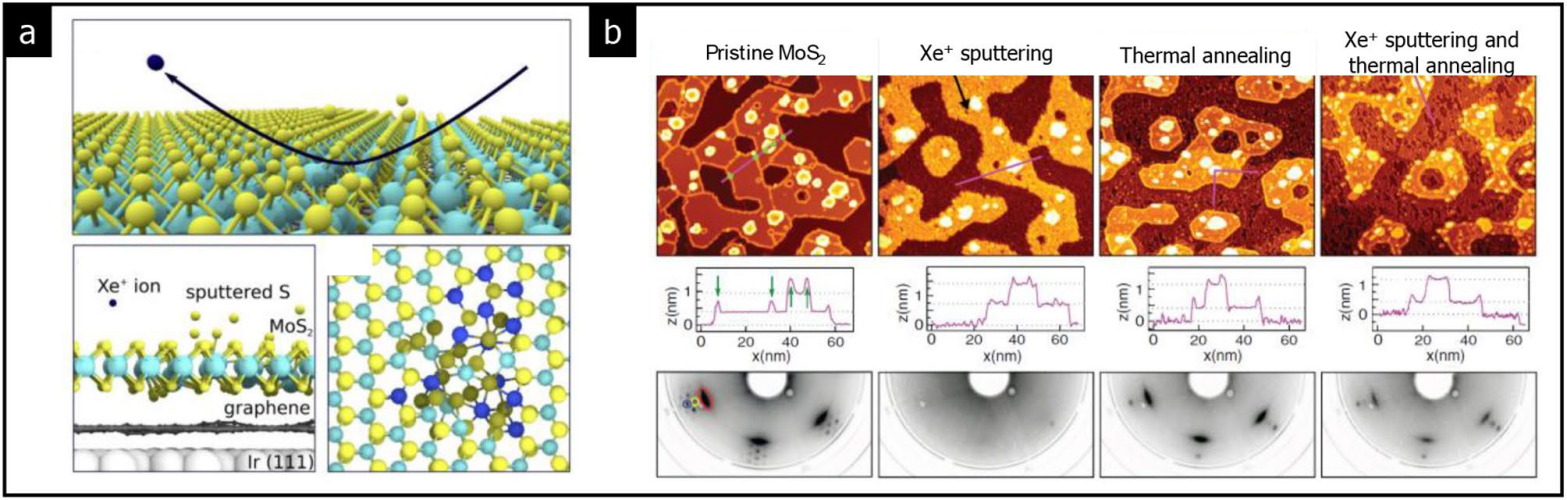
Kinetics of amorphization in MoS_2_. **a** Amorphization of MoS_2_ by impinging Xe^+^ ions on the surface of MoS_2_. **b** STM images and LEED patterns of MoS_2_ with subsequent Xe^+^ sputtering and thermal annealing. (Reprinted with permission from Ref [50])

Amorphization of MoS_2_ could be achieved by bombarding its surface with Xe^+^ ions, resulting in the sputtering of S ions, as shown in Fig. 3(a) [50]. When Xe^+^ ions glancingly impact the MoS_2_ surface, sulfur atoms are ejected from the top sulfur layer of MoS_2_ without causing damage to the underlying graphene and Ir (111) substrate. Subsequently, thermal annealing in a sulfur vapor environment facilitates recrystallization as shown in Fig. 3(b). The low-energy electron diffraction (LEED) patterns of MoS_2_, indicated by red circles, vanish following the Xe^+^ sputtering process and reappear after thermal annealing. This observation confirms that the phase transition between crystalline and amorphous phases occurs reversibly; sulfur atoms are detached during sputtering and reattached during thermal annealing.

**Fig. 4:**
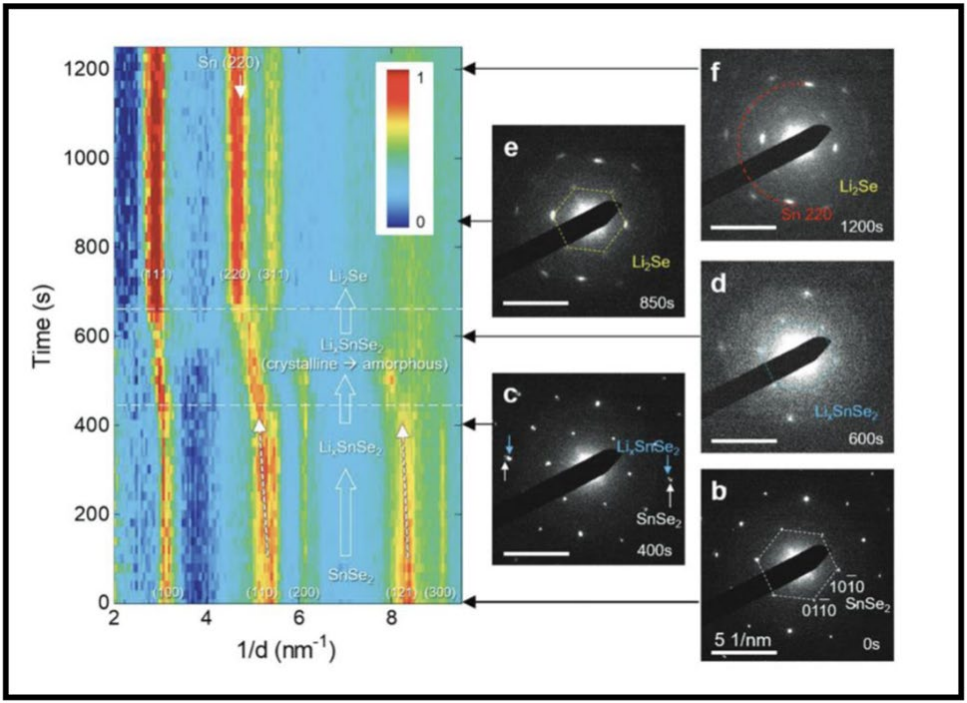
Intercalation-induced amorphization in crystalline SnSe_2_. (Reprinted with permission from Ref [51])

Another method to induce amorphization is by intercalating 2D materials with alkali atoms/ions. Figure 4 illustrates that SnSe_2_ undergoes a reversible crystalline-amorphous transition via lithiation and delithiation processes involving excess Li^+^ ions [51]. They claimed that Li^+^ ions can insert into the SnSe_2_ lattice, forming amorphous Li_x_SnSe_2_. As the concentration of Li^+^ in SnSe_2_ increases, Li_x_SnSe_2_ decomposes into Li_2_Se and Sn.

**Fig. 5:**
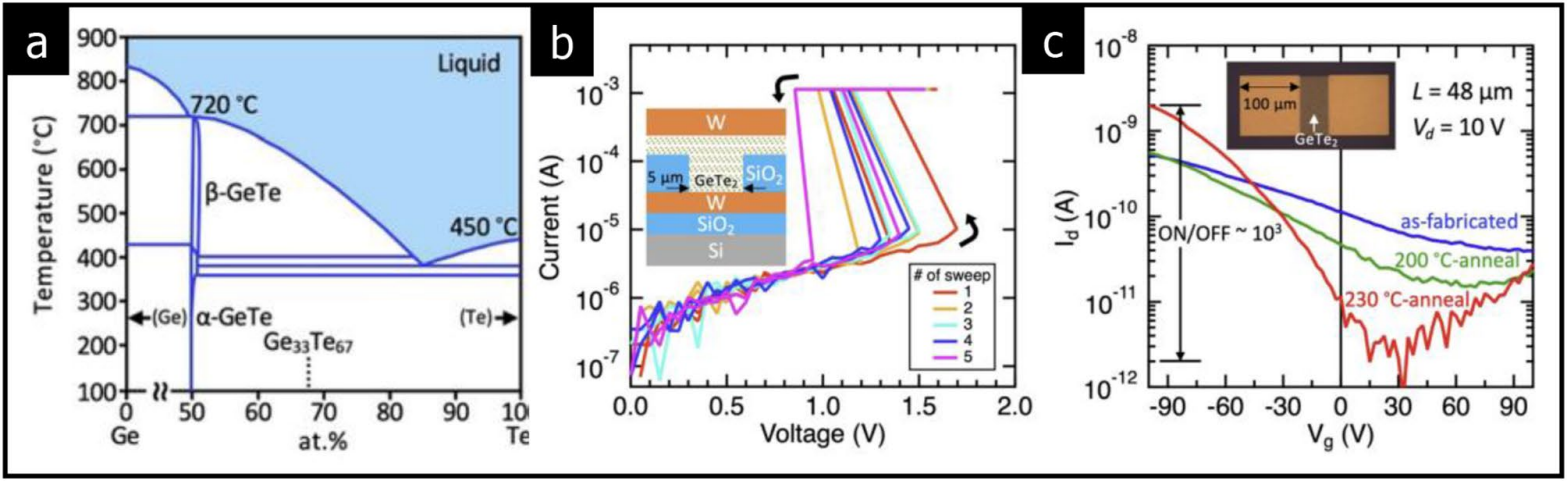
Metastable amorphous phase in the Ge-Te system. **a** Phase diagram of Ge-Te. **b** Nonlinear I-V curves in transport. **c** Enhanced device performance (on/off ratio) after additional thermal annealing. (Reprinted with permission from Ref [52])

Research has revealed phase transitions that are not represented in traditional phase diagrams. Metastable amorphous and crystalline phases can be achieved in layered geometries, offering unique opportunities for various device applications. For instance, although layered GeTe_2_ is classified as a metastable phase in the Ge-Te phase diagram (see Fig. 5(a)), Saito et al. reported a phase transition between amorphous and metastable crystalline phases while maintaining a stoichiometric ratio of 1:2 for Ge and Te elements [52].

The nonlinear I-V characteristics illustrated in Fig. 5(b) can be attributed to Ovonic threshold switching, a phenomenon commonly observed in many amorphous solids [53, 54]. Furthermore, as shown in Fig. 5(c), additional thermal annealing of the as-fabricated amorphous Ge-Te devices significantly enhances the on/off ratio by reducing contact resistance.

## Amorphous film fabrication

**Chapter 3** introduces the methods for fabricating large-scale amorphous solids, primarily in thin film geometry. The methods discussed include physical vapor deposition (PVD), chemical vapor deposition (CVD), and various modified techniques. Among these, PVD is notable for its precision and control for 2D amorphous solids, utilizing a synthetic method to deposit vapor atoms that are physically released from target materials. Various PVD techniques are employed based on the mechanisms used to generate vapor atoms and deposit them on a substrate, including Pulsed Laser Deposition (PLD), Sputtering, Molecular beam epitaxy (MBE), Thermal evaporation, and E-beam evaporation. PVD is advantageous due to its fast growth rates and the ability to control film thickness down to atomic scale.

CVD plays a crucial role in the semiconductor industry, particularly with the recent success of metal-organic CVD (MOCVD), which has demonstrated the ability to synthesize atomically thin and crystalline semiconducting materials, such as MoS_2_, on a wafer scale. Conventional CVD processes typically utilize various forms of precursors to achieve highly crystalline phases of materials at the lowest possible temperature. However, this chapter focuses on efforts to obtain amorphous thin films using CVD techniques.

### Physical vapor deposition (PVD)

PVD methods typically consist of three key steps: (1) vaporizing the target solids at high temperature in a vacuum, (2) transferring the vapor atoms or molecules to the substrate surface, and (3) depositing the vapor atoms or molecules onto the substrate to form a thin film [55]. Maintaining a high vacuum condition in the deposition chamber is critical during this process, as it allows for a long mean-free path of the source material, enabling efficient movement of vapor particles to the substrate [56].

**Fig. 6:**
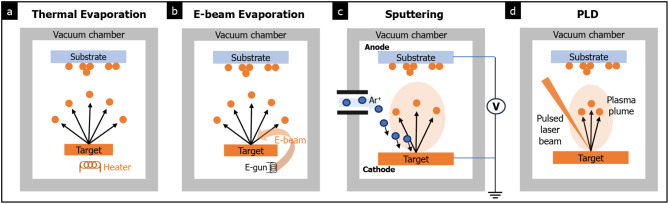
Various PVD methods using vacuum chambers. **a** Thermal evaporation **b** E-beam evaporation **c** Sputtering **d** PLD

In thermal evaporation, the target solids in crucibles are evaporated using a resistive wire heated in a vacuum chamber (see Fig. 6(a)). This method provides a high growth rate and a clean environment for fabricating 2D amorphous solids. However, it is essential to be aware that contaminants in the crucibles can inadequately deposit in the thin film, potentially degrading the performance of the materials. Despite this limitation, an amorphous Se-alloyed Te–TeO_x_ thin film has been successfully synthesized using thermal evaporation followed by additional thermal treatment [57]. The resulting amorphous Se-alloyed Te–TeO_x_ was characterized as a p-type semiconductor with high carrier mobility.

In electron beam (e-beam) evaporation, electrons are employed to heat the crucible. In a vacuum chamber (see Fig. 6(b)), electrons are generated by a heated filament (i.e., an electron emitter). These electrons are then accelerated to high kinetic energies, reaching up to tens of kV, as they move towards the crucible, where a high electric voltage is applied between the crucible and filament. This e-beam irradiation significantly elevates the temperature of the target solids in the crucible, often exceeding T = 2000 K, leading to sublimation in the vacuum chamber. The growth rate in e-beam evaporation typically ranges from 0.1 to 100 μm/min, which is higher than other PVD methods [56].

In sputtering, ions or molecules are generated from target solids through the bombardment of charged inert gas atoms in a vacuum chamber. These ions or molecules are then accelerated and deposited onto substrates (see Fig. 6(c)). It has been reported that amorphous MoTe_2 − x_ films can be synthesized using radio frequency (RF) sputtering with a MoTe_2_ target [14]. Following the sputtering process, a subsequent thermal annealing process is required to sublimate excess Te from the as-synthesized MoTe_2 − x_ films, converting them into amorphous solids. These amorphous MoTe_2 − x_ films can be transformed back into crystalline phases, 1T’ and 2 H, through further thermal annealing.

Additionally, amorphous FeSn_1 − x_ (0.42 ≤ x ≤ 0.87) films with thicknesses ranging from 28 to 46 nm have been successfully synthesized by co-sputtering Fe and Sn sources, resulting in films devoid of crystalline domains [58]. These amorphous Fe-Sn films exhibit significant anomalous Hall and Nernst effects, comparable to those observed in their crystalline counterparts.

PLD has been extensively utilized for the synthesis of complex oxide thin films, superlattices, and heterostructures. In PLD, a target solid is locally evaporated by pulsed laser irradiation to form a plasma plume, from which atoms and molecules are subsequently deposited onto a substrate (see Fig. 6(d)). Unlike sputtering, which relies on ion bombardment to evaporate the target solids, PLD uses a pulsed laser to generate a plasma plume by sublimating, evaporating, and melting the target materials.

This highly activated plasma plume allows for the deposition of thin films onto various substrates at relatively low temperatures. Additionally, the laser parameters can be easily adjusted to control the thickness and quality of the resulting amorphous thin films. However, it is worth noting that PLD often induces an island-nucleation-growth mode, particularly in the ultra-thin thickness range.

Glavin et al. demonstrated that a heterostructure of amorphous BN and amorphous MoS_2_ can be grown on a polydimethylsiloxane (PDMS) substrate at low temperatures below 200 °C using the PLD process [20]. They noted that the dielectric constant of the PLD-grown amorphous BN films is retained across a wide thickness range of 2 to 600 nm, in comparison to crystalline BN films. However, for ultra-thin amorphous BN films with thicknesses less than 2 nm, there is a significant decrease in dielectric strength, accompanied by non-uniform thickness in the PLD-grown material.

The properties of amorphous thin films, including crystal structures, morphologies, and film qualities, are highly sensitive to growth conditions [59, 60]. Nagarajan et al. reported that a non-stoichiometric amorphous GaO_x_ with excess Ga can be synthesized in a reducing atmosphere, whereas stoichiometric transparent Ga_2_O_3_ is formed in an oxidizing atmosphere [61]. Additionally, Kim et al. found that PLD can produce an amorphous Ga_2_O_x_ thin film on a glass substrate at room temperature, and that the gas environment during post-annealing significantly affects the crystal structures and chemical compositions of the films [62].

### Chemical vapor deposition (CVD)

**Fig. 7:**
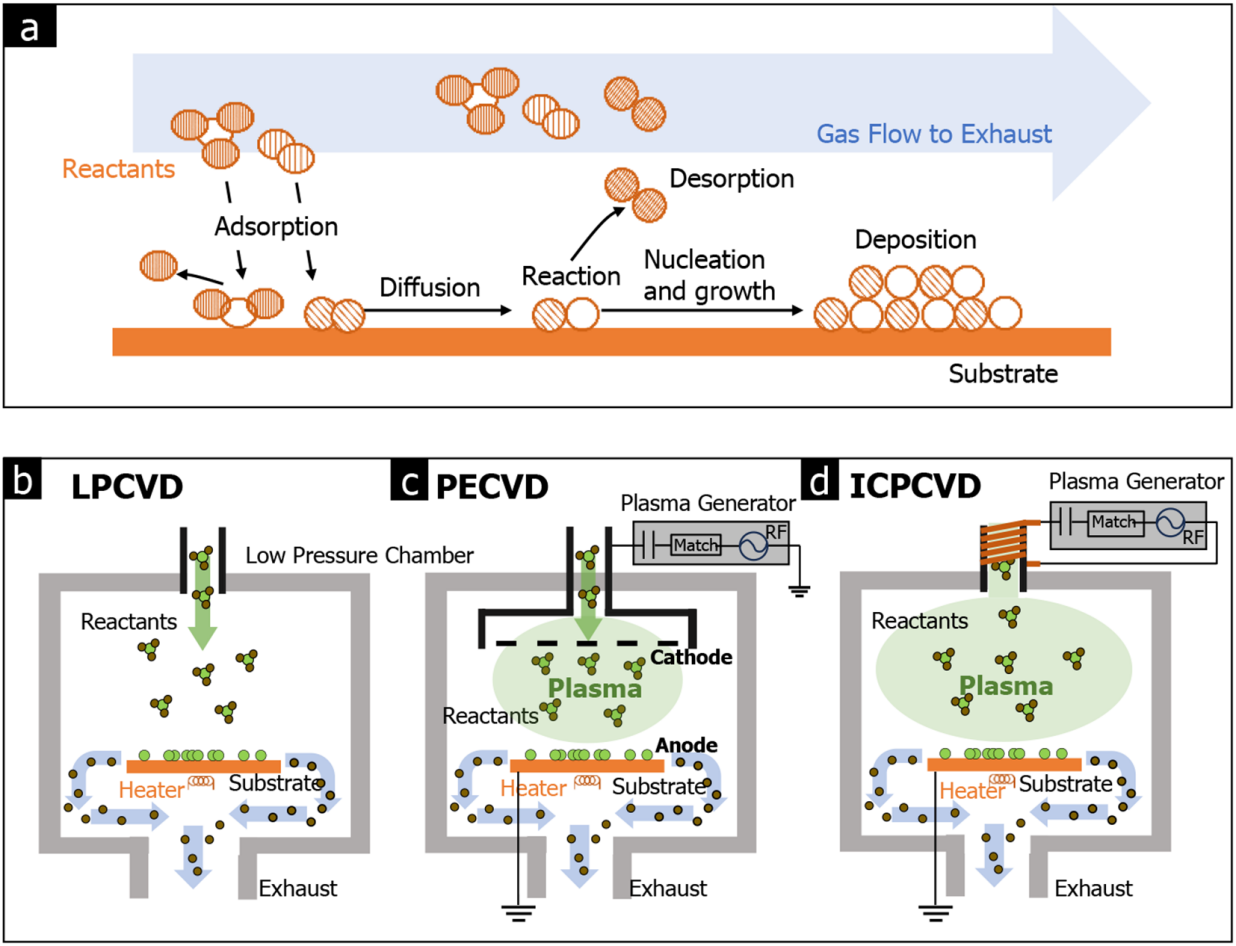
Various types of CVD methods. **a** Growth mechanism of CVD. **b** LP(Low-pressure)CVD. **c** PE(Plasma-enhanced)CVD. **d** ICP(Inductively coupled plasma)CVD

The low-pressure CVD (LPCVD) processes offer advantages in achieving high purity and uniformity in the synthesized films (see Fig. 7(a)). However, the reduced number of gas molecules in LPCVD leads to a lower growth rate, whereas the high-pressure CVD process tends to exhibit the opposite trend, with a higher growth rate but lower uniformity. Maintaining a uniform temperature during the growth process is crucial in low-pressure conditions. To address this, a horizontal tube furnace (reactor) is specially designed for LPCVD, as illustrated in Fig. 7(b) [63].

It has been reported that amorphous Si and its derivatives can be synthesized using LPCVD at temperatures slightly below the crystallization point. Similarly, amorphous BN films have been successfully synthesized at T = 500 °C using LPCVD. Recent studies highlight the promising properties of amorphous BN, which show excellent passivation and heat dissipation capabilities, leading to enhanced device performance. These improvements include reduced current fluctuations and a tenfold increase in carrier mobility [64].

In addition, centimeter-scale uniform amorphous BN films have been synthesized using LPCVD in a sandwich structure, where top and bottom amorphous BN layers encapsulate graphene. This structure could be directly applied in high-performance graphene-based field-effect transistors [65].

High-temperature synthesis using LPCVD can induce significant tensile stress in amorphous solids, which can be advantageous for nano-mechanics applications [62–67]. Xu et al. reported that the LPCVD-grown amorphous SiC exhibits high chemical inertness against commonly used dry etchants, facilitating nano-fabrication across various substrates [66]. The authors further noted that wafer-scale amorphous SiC films synthesized via LPCVD at high temperatures demonstrate impressive tensile strength, exceeding 10 GPa.

When synthesizing amorphous solids using CVD at lower temperatures, additional energy sources are often necessary to drive the chemical reactions. One such method involves the use of plasma, known as plasma-enhanced CVD (PECVD), depicted in Fig. 7(c) [68]. Non-stoichiometric amorphous BN films synthesized by PECVD offer easy mechanical exfoliation, similar to crystalline van der Waals (vdW) 2D materials, enabling the fabrication of vertical heterostructures with amorphous solids [69].

Inductively coupled plasma CVD (ICPCVD) (see Fig. 7(d)) is a modified CVD method that utilizes a higher plasma density compared to conventional PECVD [70]. ICPCVD enables the growth of high-quality amorphous films at low temperatures, even as low as room temperature. Using ICPCVD, various amorphous solids, such as amorphous Si, SiO_2_, and SiC films, have been successfully synthesized at low temperatures [71–74].

Recently, atomically thin amorphous BN films grown via ICPCVD have been reported, exhibiting an ultra-low dielectric constant, exceptional mechanical strength, and high thermodynamic stability. These properties make them highly suitable for diffusion barriers in nanoelectronics metal interconnects [13, 75].

Compared to CVD, atomic layer deposition (ALD) is a seed-free synthesis method that produces amorphous solids with fewer defects and dislocations caused by nucleation faults. ALD also enables precise control of thickness and chemical composition at the atomic level. Recently, wafer-scale, uniform amorphous BN films have been grown using low-temperature ALD. Additionally, Wu et al. synthesized amorphous MoS₂ films on a glassy carbon substrate at low temperatures using plasma-assisted ALD [76]. Their findings revealed that the amorphous MoS_2_ films exhibit shorter Mo-Mo and Mo-S bonds compared to their crystalline counterparts, which is the key to their modified physical and chemical properties.

ALD has also been utilized to synthesize atomically thin amorphous Al₂O₃ films, enabling the precise fabrication of vertical heterostructures with amorphous solids. These structures are suitable for memristive device applications [77].

### Thermal conversion

**Fig. 8:**
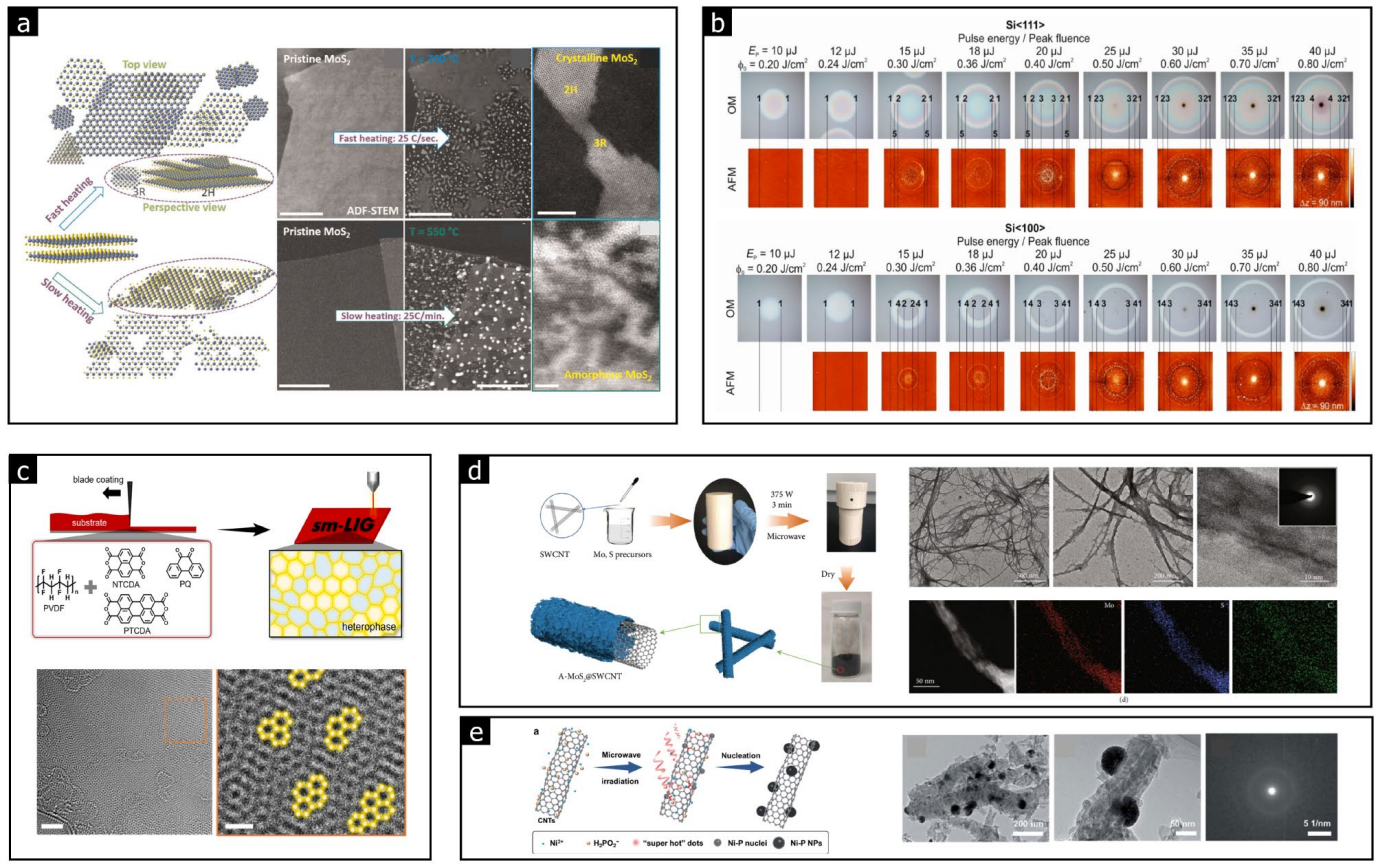
Thermal conversion to amorphous phases. **a** Fast and slow heating in MoS_2_ (Reprinted with permission from Ref [78]. **b** Laser-induced amorphization of Si with different crystal orientations (Reprinted with permission from Ref [79]. **c** Conversion of small molecules into graphene with a tunable amorphous/crystalline heterophase structure by IR laser irradiation (Reprinted with permission from Ref [82]). **d** Solution-based synthesis of amorphous MoS_2_ on CNTs using microwave heating (Reprinted with permission from Ref [83]). **e** Amorphous NiP on CNTs synthesized using microwave irradiation (Reprinted with permission from Ref [84])

Thermal treatment is an effective method for introducing significant disorder into crystal structures. Kumar et al. demonstrated that when crystalline MoS_2_ is slowly heated, it decomposes into amorphous Mo_x_S_2−x_ due to sulfur evaporation [78]. Two distinct thermal treatments were applied to MoS_2_: one under non-equilibrium conditions with rapid heating and another under equilibrium conditions with slow heating. As shown in Fig. 8(a), amorphous MoS_2_ can be produced under slow heating conditions. However, this method may result in decomposition due to substantial changes in stoichiometry.

Thermal conversion through localized heating using laser or microwave irradiation offers an effective strategy for synthesizing 2D amorphous solids while preserving material properties and ensuring phase stability. Laser irradiation enables localized heating by providing sufficient thermal energy for amorphization [79–81]. Florian et al. found that laser fluence is critical in the amorphization of Si, and this effect varies with crystal orientations (see Fig. 8(b)) [79]. Cheng et al. demonstrated that infrared (IR) laser irradiation can convert aromatic molecules into a graphene-like structure, featuring a mixture of amorphous and crystalline phases [82]. This graphene-like structure consists of randomly arranged hexagonal nanocrystals and carbon polygons, with the crystalline-to-amorphous phase ratio depending on the laser power and pulse density (see Fig. 8(c)).

Laser irradiation induces localized thermal treatment with a specific temperature distribution, causing non-uniform thermal conversion to the amorphous phase over a broad area. In contrast, microwave irradiation generates large-scale atomic vibrations that rapidly produce significant thermal energy without convection or diffusion processes. This simplifies the synthesis of amorphous solids by altering atomic structures, breaking chemical bonds, and activating atoms or molecules to promote chemical reactions.

Tang et al. reported the successful amorphization of MoS_2_ on carbon nanotubes (CNTs) via microwave irradiation [83]. As shown in TEM images (see Fig. 8(d)), the amorphous MoS_2_ was uniformly formed on the CNT surface without aggregation. Similarly, amorphous NiP nanoparticles were synthesized on CNTs through a one-pot microwave process (see Fig. 8(e)) [84].

## Applications for sub-nanometer scale devices

### Gate dielectrics and electric insulator

For several decades, high-dielectric (κ) amorphous solids have been widely utilized as key components in capacitors for semiconducting devices. In addition, 2D amorphous dielectric solids have shown significant promise as ultra-thin dielectric layers in nanometer-scaled electronics. Despite their potential, wide-gap, high-κ, layered dielectric solids with vdW structures have been less explored and rarely used. This is primarily due to the significant leakage current that occurs through thin and amorphous dielectric layers [85].

**Fig. 9:**
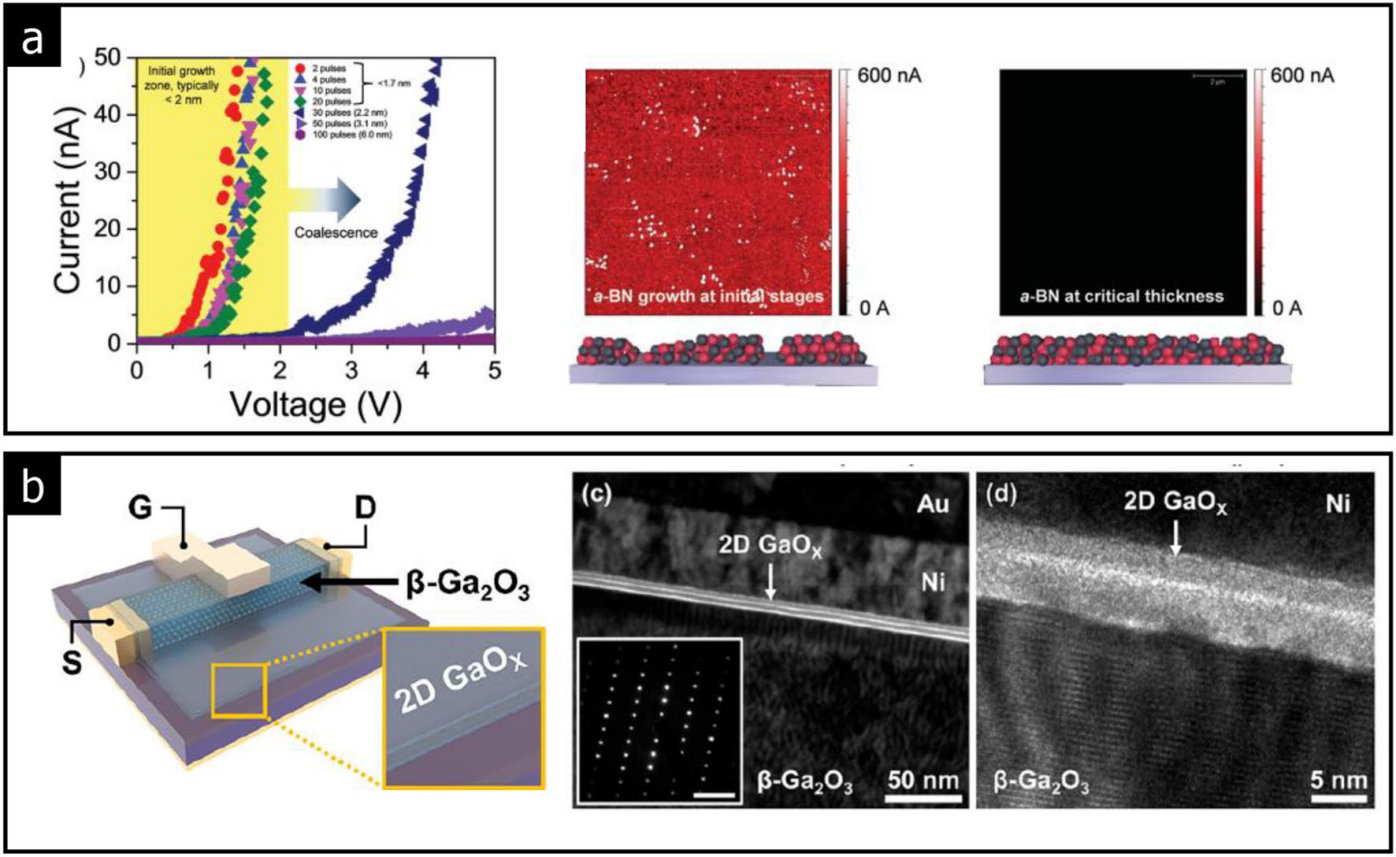
High dielectric (κ) amorphous solids. **a** I– V curves of amorphous BN with different thicknesses controlled by the number of laser pulses during PLD growth (Reprinted with permission from Ref [20]). **b** Schematics and cross-sectional TEM images of a 2D GaO_x_/β-Ga_2_O_3_ MISFET (Reprinted with permission from Ref [86])

Glavin et al. reported that amorphous BN with thicknesses from 2 to 17 nm exhibits a dielectric constant of 5.9 at 1 kHz, a breakdown field of 9.8 MV/cm, and a bandgap of 4.5 eV, which are comparable to those of crystalline BN [20]. However, when the thickness of amorphous BN is reduced to below 2 nm, the growth mechanism shifts to island nucleation. This results in dielectric breakdown at lower voltages, as illustrated in Fig. 9(a).

2D amorphous gallium oxide (GaO_x_) has been synthesized using the liquid-gallium squeezing technique (see Fig. 9(b)) [86]. The 2D GaO_x_ bilayer, with a thickness of 8.5 nm, exhibits a dielectric constant of 6.5 and the dielectric breakdown voltages of ± 6 V. A metal-insulator-semiconductor field effect transistor (MISFET) was fabricated using metallic Ni, insulating amorphous GaO_x_ as the dielectric, and a semiconducting crystalline ß-Ga_2_O_3_ channel. This device demonstrated excellent performance at the atomic scale, achieving a high on/off ratio of 5.3 × 10^8^ and field-effect carrier mobility of 6.8 cm^2^∙V^− 1^∙s^− 1^.

**Fig. 10:**
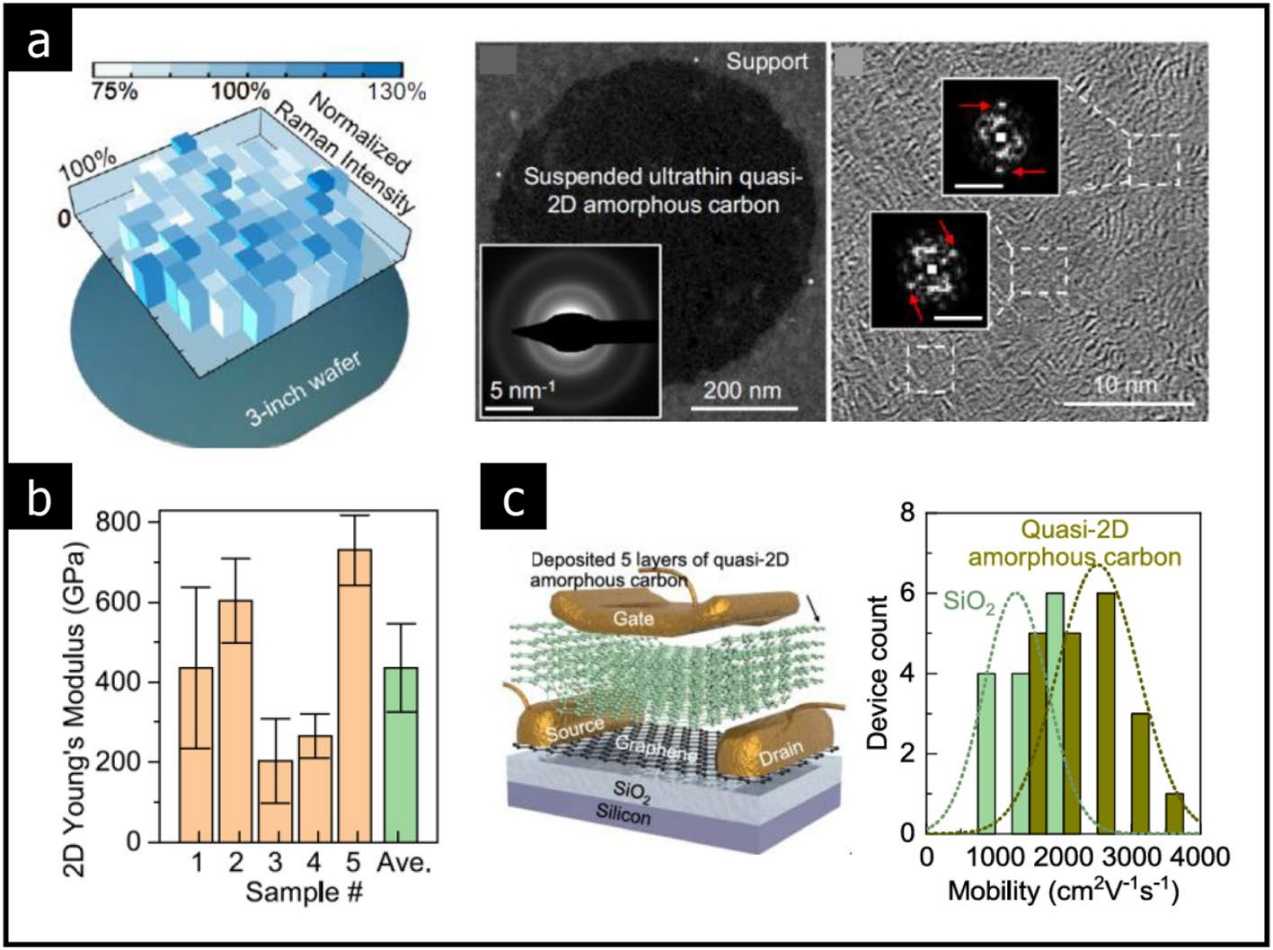
Graphene transistor with ultrathin 2D amorphous carbon dielectric layers. **a** Atomically thin 2D amorphous carbon film deposited on a 3-inch wafer. TEM images and SAED patterns of ultrathin amorphous carbon film. **b** 2D Young’s moduli of ultrathin amorphous carbon films. **c** Graphene transistor with five layers of amorphous carbon as the top-gate dielectric. The field-effect mobility of bottom-gated graphene device with SiO_2_ gate oxide and top-gated graphene device with amorphous carbon as a dielectric. (Reprinted with permission from Ref [11])

Wafer-scale ultrathin 2D amorphous carbon has been synthesized from solution precursors, achieving thicknesses down to 1–2 atomic layers, as shown in Fig. 10(a) [11]. The amorphous carbon exhibits remarkable mechanical strength, with Young’s modulus of 500 GPa (see Fig. 10(b)). Additionally, a graphene transistor has been fabricated utilizing amorphous carbon dielectrics, as depicted in Fig. 10(c). The 2.4 nm-thick (5 layers) amorphous carbon demonstrates high capacitance, which enhances the electrostatic coupling between the gate electrode and the graphene channel. The macroscopic uniformity and low surface dangling bonds in the amorphous carbon layers contribute to excellent device performance, achieving a high carrier mobility of 2000–3000 cm^2^∙V^− 1^∙s^− 1^, a low leakage current density of 10^− 4^ A/cm^2^, and a breakdown field of 20 MV/cm.

In summary, high-dielectric amorphous solids with large bandgap are emerging as viable gate dielectrics in atomic-scale electronic devices. It is important to note that the dielectric constant of conventional 2D solids tends to decrease as the sample thickness diminishes, primarily due to the significant suppression of cross-plan polarization at the atomic scale [87, 88]. Therefore, while the thickness of amorphous gate dielectric can be reduced to several atomic layers (≥ 2 nm), there remains a limitation for effective operation at scales below 1 nm in high-κ dielectrics.

In recent years, the development of highly integrated nanometer-scaled electronics has sparked significant interest in ultralow dielectric constant amorphous solids. These materials are crucial for simultaneously reducing both resistance and capacitance in the devices. Additionally, ultralow dielectric amorphous solids facilitate electrical isolation at the nanometer scale while offering high mechanical strength.

**Fig. 11:**
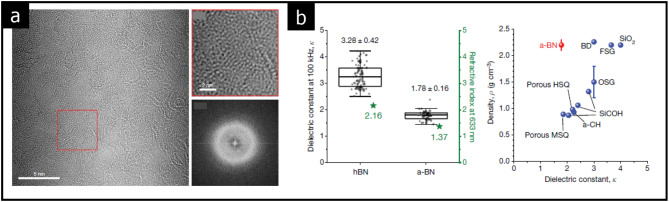
Ultralow dielectric amorphous BN. **a** TEM images of amorphous BN. **b** Dielectric constants of amorphous and crystalline BN and their density with reference crystals. (Reprinted with permission from Ref [13])

Hong et al. reported that a 3 nm-thick amorphous BN film exhibits an ultralow dielectric constant close to that of air (k = 1) (see Fig. 11 (a) and (b)) [13]. The amorphous BN demonstrates impressive mechanical properties, with a Young’s modulus exceeding 11 GPa, and exceptional electronic properties, featuring a breakdown field of 7.3 MV/cm. Furthermore, the authors noted that the density of amorphous BN ranges from 2.1 to 2.3 g/cm^3^, which is comparable to that of crystalline BN, 2.1 g/cm^3^(see Fig. 11 (b)). Thus, 2D amorphous insulating solids with both high and low dielectric constants present promising applications as gate dielectrics and for electric or thermal isolation in nanoelectronics.

### Semiconducting channels in field effect transistors

In general, crystalline solids tend to exhibit higher conductivity than amorphous solids, primarily because structural disorders in amorphous structures act as scattering centers. Nevertheless, some amorphous solids demonstrate relatively high conductivity due to factors such as shallow donor/acceptor levels, efficient electron hopping across the conducting areas, continuous in-gap states, and specific topological features. Below, we briefly review recent studies that report the conducting behaviors of amorphous solids with 2D geometry.

**Fig. 12:**
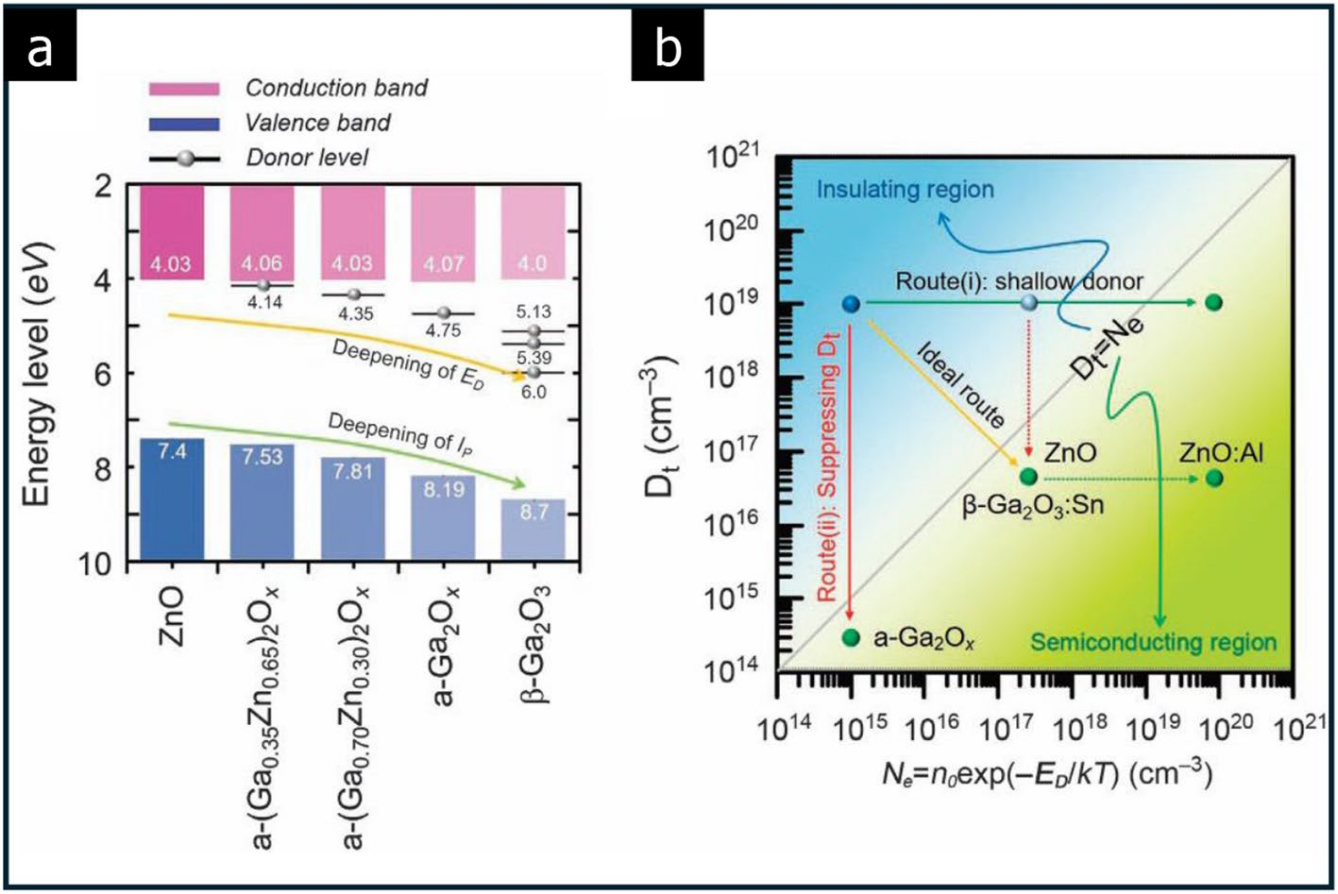
Insulator-to-semiconductor transition in amorphous Ga-Zn-O. **a** Energy levels of amorphous solids with various donor levels. **b** Transition between insulating and semiconducting phase as a function of electron trap density (D_t_) and electron density (N_e_). (Reprinted with permission from Ref [62])

Several reports have highlighted transitions between insulating and (partially conducting) semiconducting phases in amorphous solids, driven by modulating electron trap density. These amorphous materials often exhibit variable stoichiometric ratios between their constituent elements. For instance, as shown in Fig. 12(a), the energy diagram and carrier density of the crystalline and amorphous Ga-Zn-O series depend on the varying contents of Zn and Ga. Thus, the bandgap can be modulated from 3.37 to 4.70 eV [62].

Defect levels arise from substitutional Ga and Zn atoms, allowing for exploration of the phase diagram as a function of electron trap density (D_t_) and electron density (N_e_). A transition from an insulating to a semiconducting phase can be achieved either by suppressing D_t_ (in Route (i)) or by creating shallow donor levels (in Route (ii)) in the amorphous Ga-Zn-O series (Fig. 12(b)).

**Fig. 13:**
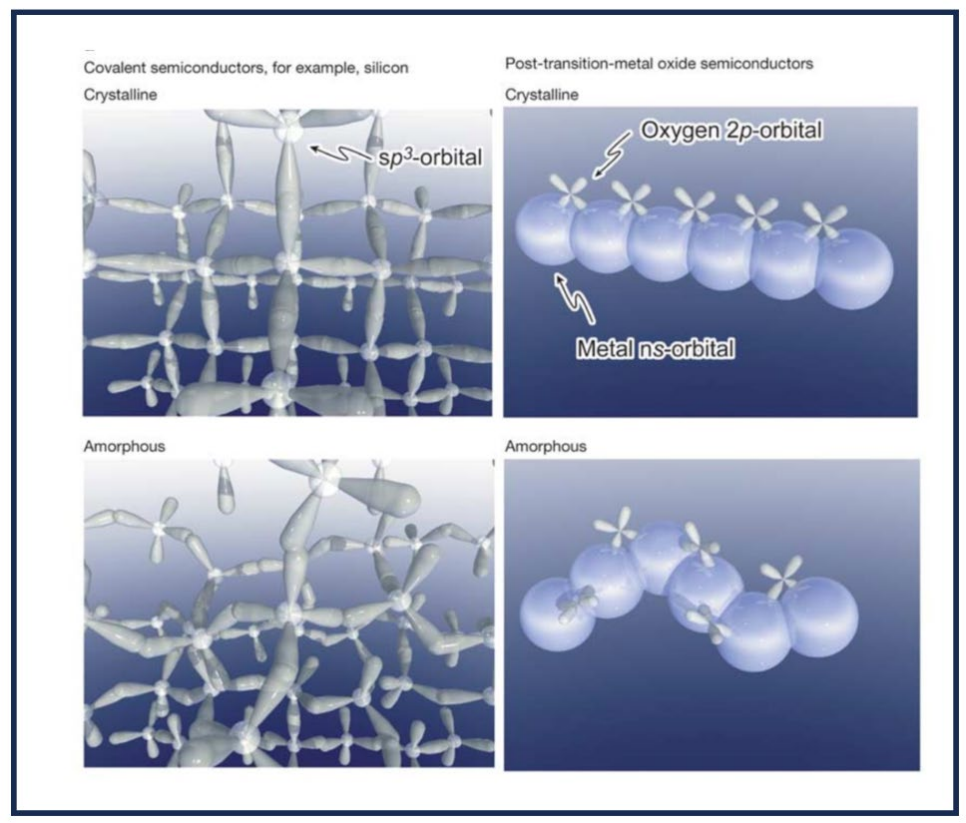
Carrier transport paths of crystalline Si and amorphous transition-metal oxide, depicted with electron orbitals. (Reprinted with permission from Ref [89])

The orbital topology also plays a crucial role in enhancing carrier mobility in amorphous solids. As illustrated in Fig. 13, carrier transport paths differ significantly between crystalline Si and amorphous transition-metal oxide [89]. In crystalline Si, the strong directional nature of sp^3^-orbitals means that atomic bond overlaps are highly sensitive to structural disorders, which leads to a reduction in carrier mobility in its amorphous form. Conversely, in transition-metal oxides, the conduction bands are predominantly formed by spherically symmetric s-orbitals.

This symmetry makes atomic bond overlaps less susceptible to structural disorders, allowing amorphous transition-metal oxides to retain high mobility even in disordered states. Accordingly, transparent amorphous oxide semiconductors exhibit Hall effect mobilities of around 10 cm^2^·V^− 1^·s^− 1^, which is significantly higher than the 1 cm^2^·V^− 1^·s^− 1^ mobility typically observed in amorphous Si [89].

### Conductive metallic electrodes

**Fig. 14:**
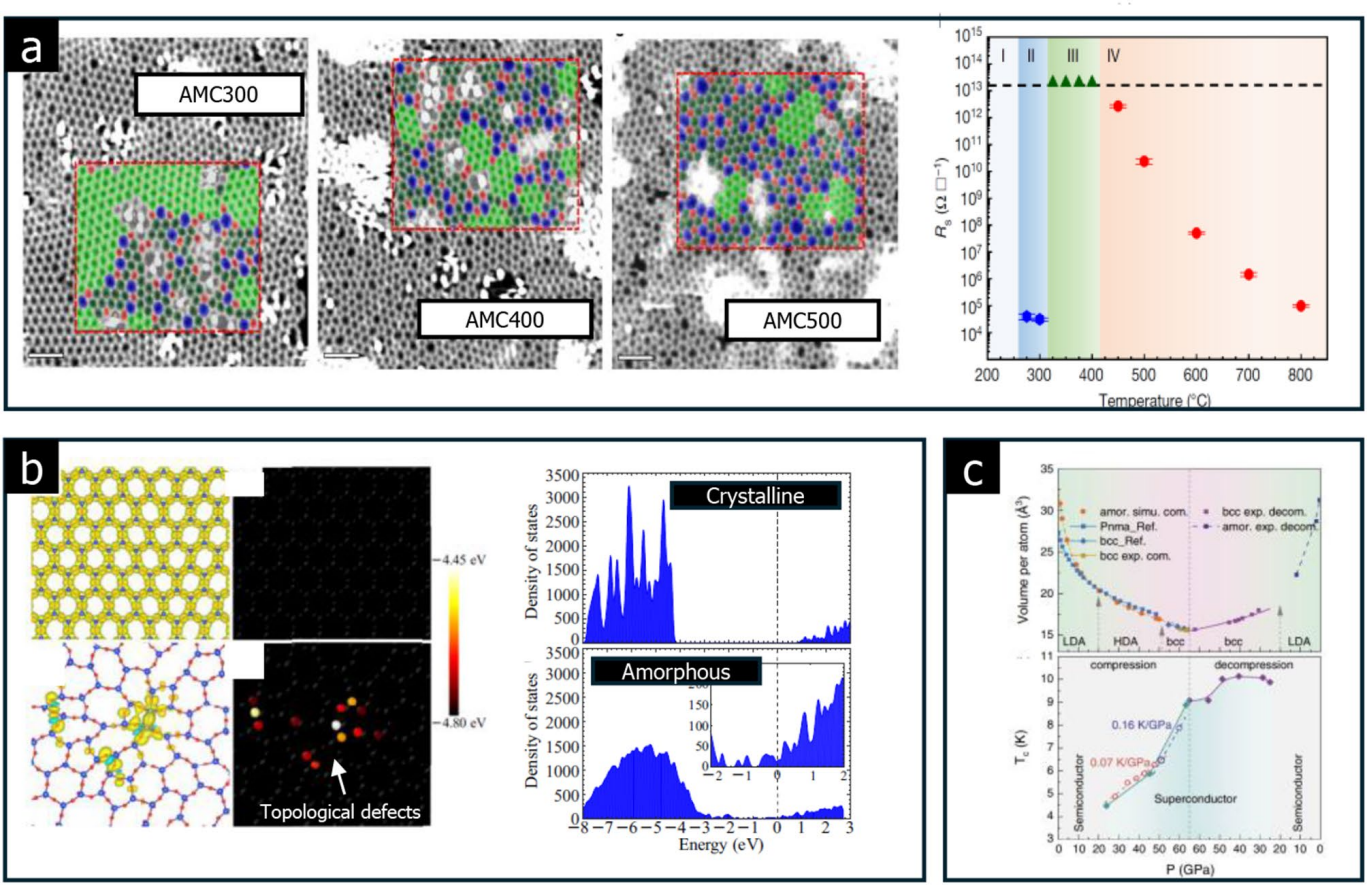
Conductive amorphous solids. **a** amorphous carbon monolayer composed of carbon rings with hexagon (green and dark green), pentagon (red), heptagon (blue), and octagon (blue) shapes. The sheet resistance (R_s_) as a function of the growth temperature (Reprinted with permission from Ref [90]). **b** Phase transition from insulator to metal, induced by disordered hyperuniformity (Reprinted with permission from Ref [91]). **c** Superconducting states in pressurized amorphous Sb_2_Se_3_ and high-density amorphous Sb_2_Se_3_ (Reprinted with permission from Ref [92])

Structural disorders can further enhance the conductivity of certain 2D amorphous solids. Tian et al. demonstrated that the growth temperatures of an amorphous carbon monolayer could modulate these structural disorders, thereby significantly influencing its electrical conductivity [90]. As depicted in Fig. 14(a), the amorphous carbon monolayer contains carbon rings of varying shapes, including hexagons (green and dark green), pentagons (red), heptagons (blue), and octagons (blue). As the growth temperature increases from 300 to 500 ^o^C, hexagon carbon rings are gradually converted into other polygon configurations. Notably, the sheet resistance (R_s_) changes dramatically around 300–325 ^o^C growth temperature range, spanning from 10^4^ to 10^13^ W∙□^−1^.

The authors suggest that electron hopping occurs between distinct regions within the amorphous carbon monolayer, which contains randomly distributed conductive crystalline and insulating amorphous areas. The size and density of these regions vary according to the growth temperature. The study highlights that such a profound change in resistance (across nine orders of magnitude) is rarely observed in bulk amorphous materials, illustrating that the 2D nature of these materials plays a crucial role in tuning their conductivity. This phenomenon shows the unique advantage of 2D amorphous solids in enhancing and controlling electrical properties through structural disorders [90].

Recently, amorphous 2D solids with atomic-scale thickness have garnered attention as a new type of 2D material with disordered hyperuniformity, which refers to a unique state where local density fluctuations are suppressed over long ranges, even in disordered structures [91]. Zheng et al. demonstrated that this disordered hyperuniformity could induce a phase transition from insulator to metal by completely closing the bandgap, as shown in Fig. 14(b). This process is distinguished from typical amorphous materials, where bandgap closure is uncommon.

There are competing theories about the metal-insulator transition in amorphous 2D solids. Some suggest Anderson localization, which emphasizes the importance of disorders and electron wave interference, while others claim that topological defects in an amorphous solid can be localized with remarkably high energies. These defects, in turn, contribute to carrier concentrations as high as 10^12^ cm^− 2^, comparable to the high doping levels found in conventional 2D semiconductors. This introduces the idea that disorder itself can drive electronic properties in amorphous 2D solids.

In a similar context, it has been reported that an amorphous MoS_2_ monolayer can undergo a semiconductor-to-metal transition. This transformation is driven by disorder- and vacancy-induced in-gap states, which provide continuous electronic pathways, leading to metallic behavior in previously semiconducting materials [50]. These insights emphasize the potential of amorphous 2D solids for advanced applications where traditional crystalline structures may not be suitable, particularly in fields where tunable conductivity is required.

High pressure is an effective tool for tuning structural disorder and electron localization in amorphous solids. Zhang et al. demonstrated this by discovering a superconducting state in pressurized amorphous Sb_2_Se_3_. At a pressure of 24 GPa, the material undergoes local structural distortion and electron localization, resulting in a high-density amorphous (see Fig. 14(c)) [92]. This highlights how external pressure can induce significant changes in amorphous solids’ electronic and structural properties, allowing for novel phases like superconductivity.

Theoretical models suggest that amorphous solids can act as topological insulators, despite lacking the spatial symmetries traditionally required for topological phases. This is due to the protection of topological phases by non-spatial symmetries in certain amorphous materials. So far, topological insulating states have been reported in several 2D amorphous solids [93, 94].

**Fig. 15:**
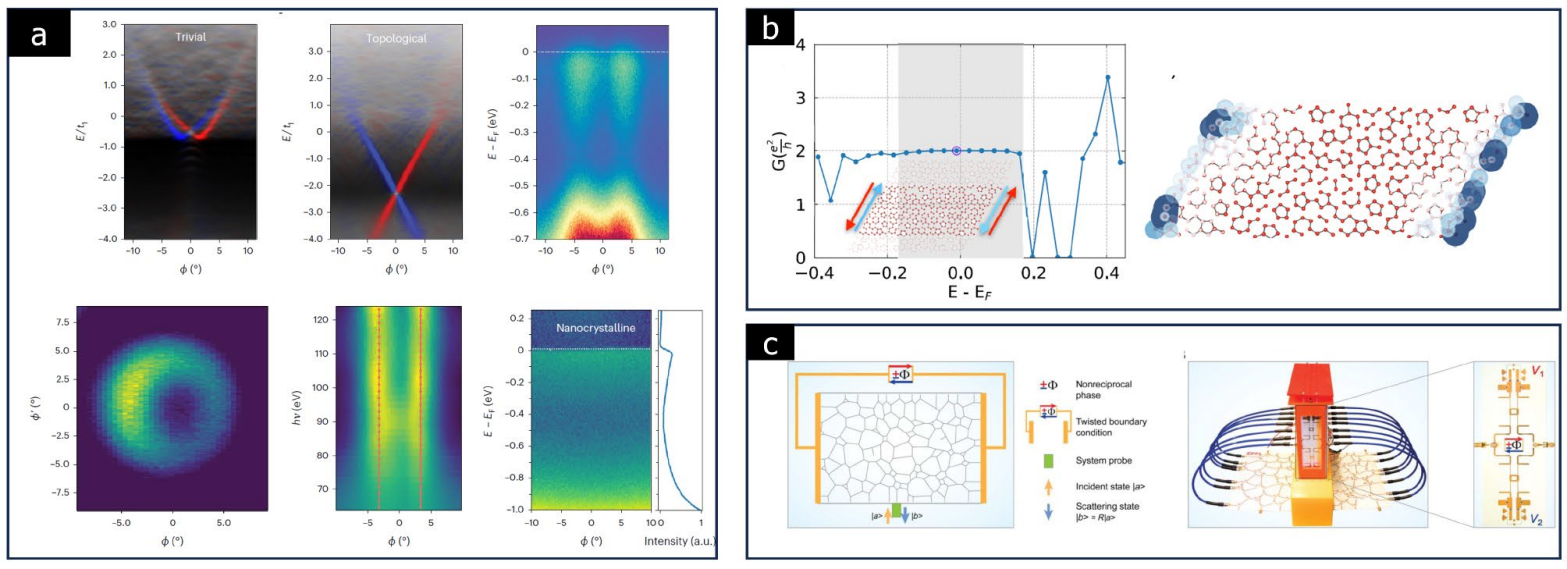
Topological behaviors in 2D amorphous solids. **a** ARPES spectra of electronic states in amorphous Bi_2_Se_3_ (Reprinted with permission from Ref [23]). **b** Two-terminal conductance of amorphous H-bismuthene nanoribbon and wave function spectral weight of a mid-gap state, projected on the lattice sites (Reprinted with permission from Ref [94]). **c** Schematic for measuring the topological features of amorphous solids and the experimental setup (Reprinted with permission from Ref [95])

Corbae et al. found that 10 nm-thick amorphous Bi_2_Se_3_ films display characteristics consistent with topological insulators. The high resistivity and weak temperature dependency of the films reflect behaviors similar to bulk Bi_2_Se_3_, which is a well-known topological insulator with a metallic surface state [23]. Furthermore, angle-resolved photoemission spectra (ARPES) measurements of amorphous Bi_2_Se_3_ film reveal a topological surface state with a Dirac cone, as shown in Fig. 15(a).

In atomically thin amorphous solids, topological metallic states can emerge at the edges, and these edge regions, with mid-gap states, contribute to the edge conductance of the materials [19, 94]. Costa et al. reported that a Bi monolayer, also known as hexagonal bismuthine, exhibits topological insulator properties based on ab initio calculations [94]. As shown in Fig. 15(b), topological conductance channels are formed at the edge of the amorphous solid, giving rise to quantized conductance observed in the quantum spin Hall effect.

Zhang et al. provided direct experimental evidence of topological edge transport in 2D amorphous solids through transmission and topological invariant measurements, as shown in Fig. 15(c) [95]. This points to the robustness of topological states, even in disordered systems like amorphous solids, where electron transport can be remarkably stable along the edges. Thus, 2D amorphous solids open new possibilities for nanoelectronics and spintronics applications with their topological states.

### Thermal insulator

Thermal conductivity is a crucial property of solids, strongly related to collective lattice vibrations, typically referred to as phonons. In crystalline solids, phonons are the primary carriers of heat, allowing coherent vibrations to transfer thermal energy across the material. However, in amorphous solids, structural disorders severely disrupt these coherent lattice vibrations, making the formation of conventional phonons difficult. Therefore, alternative mechanisms for heat transfer in amorphous solids should be explored.

**Fig. 16:**
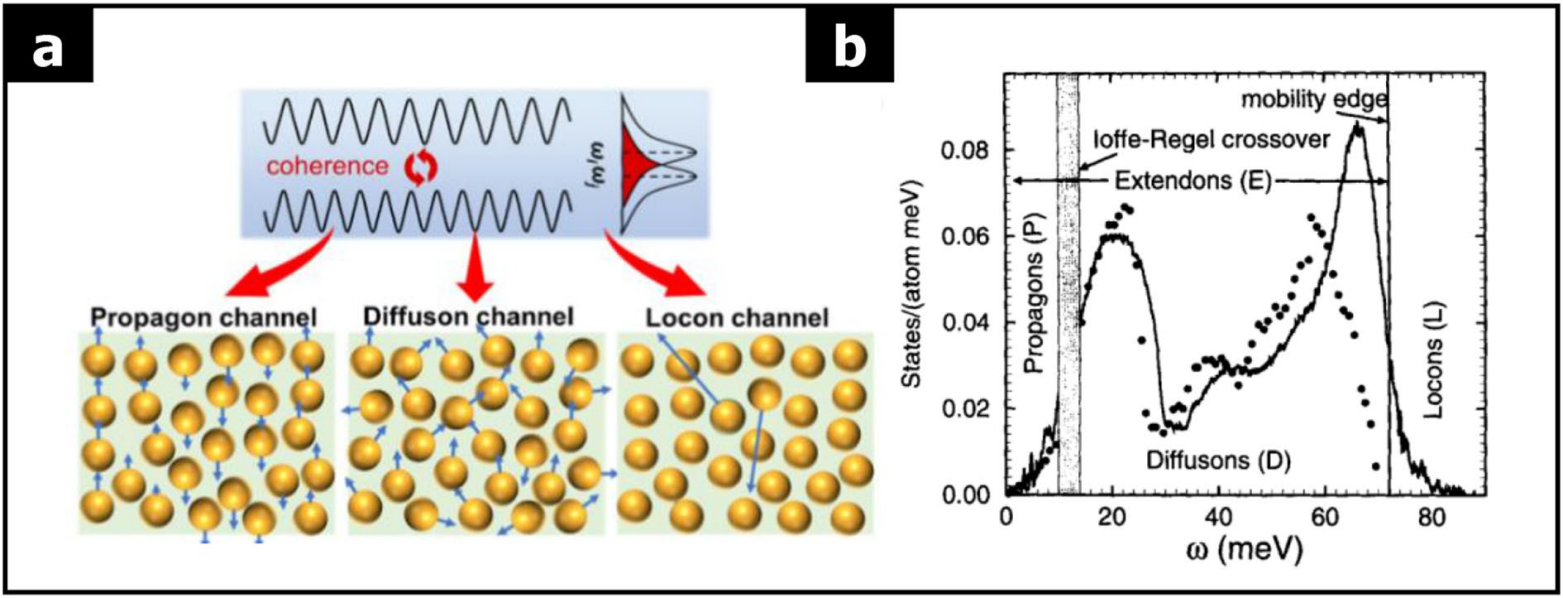
Thermal conductivity of 2D amorphous solids. **a** Schematic illustration of the multichannel thermal transport mechanism with propagons, diffusons, and locons (Reprinted with permission from Ref [96]). **b** Density of vibrational modes in amorphous Si (Reprinted with permission from Ref [97])

To explain heat transfer in amorphous solids, three distinct heat carriers have been proposed: ‘propagon’, ‘diffuson’, and ‘locon’ channels (see Fig. 16(a) [96]. These carriers represent different vibrational modes based on their frequencies and the degree of delocalization. Propagons are delocalized heat carriers that occur in the low-frequency range, behaving similarly to phonons in crystalline solids. They transfer heat through coherent vibrations. Diffusons exist in the intermediate-frequency range and contribute to heat transfer through a diffusive mechanism rather than coherent vibration, as their modes are delocalized but not as ordered as propagons. Locons are localized vibrations that occur at high frequencies, concentrated around individual atoms or clusters. Due to their localized nature, these vibrational modes contribute minimally to heat transfer.

The phonon density of amorphous Si as a function of frequency is shown in Fig. 16(b) [97, 98]. In amorphous solids, delocalized vibrational modes, like those of propagons and diffusons, can be effectively suppressed due to structural disorder. This leads to much lower thermal conductivity compared to crystalline counterparts. Understanding these heat transfer mechanisms is essential for manipulating thermal properties in amorphous solids, which is important for applications such as thermal insulation or heat management in electronic devices.

**Fig. 17:**
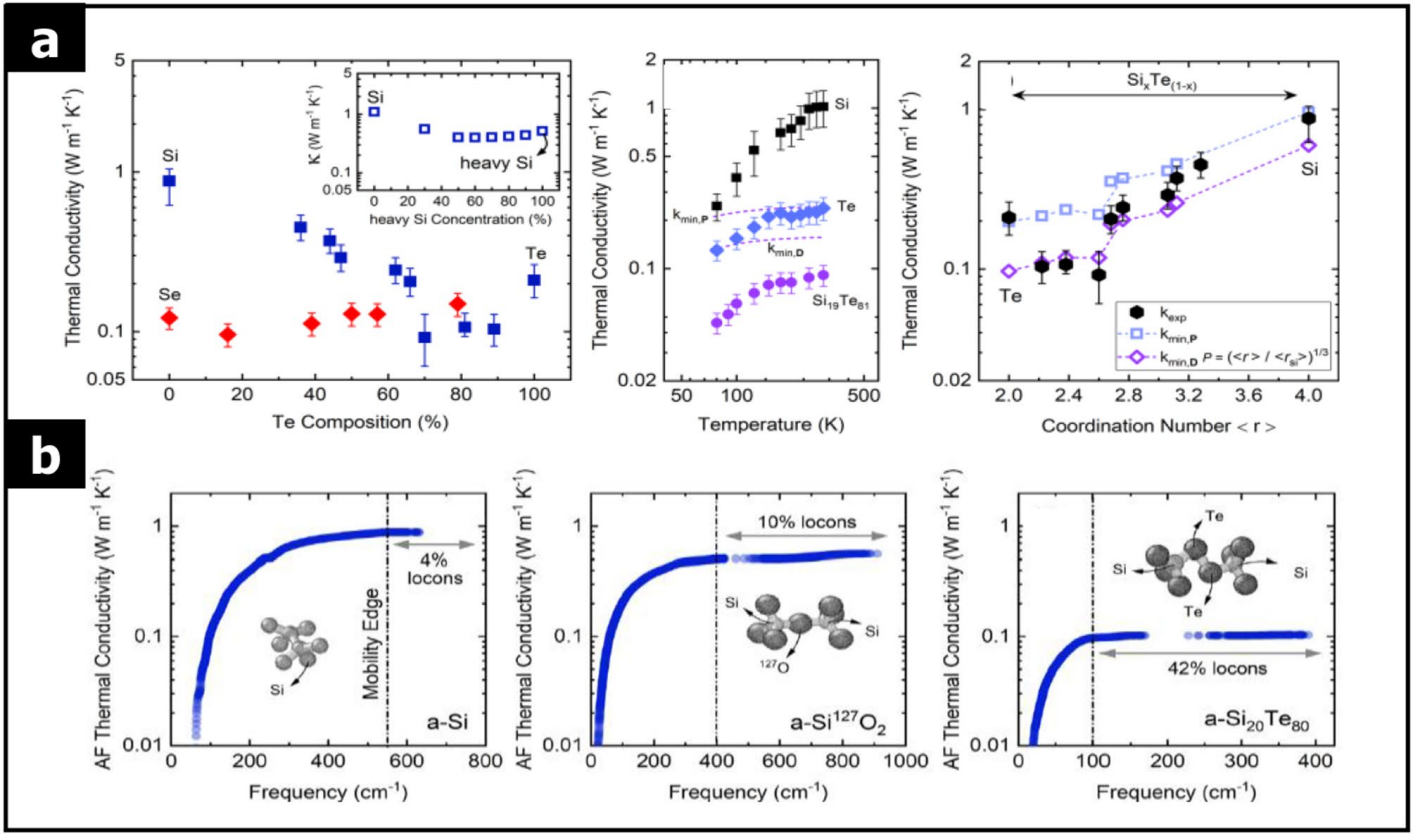
Thermal properties of 2D amorphous solids. **a** Thermal conductivity of amorphous Si-Te as a function of Te composition and thermal conductivity of Si_1 − x_Te_x_ as a function of coordination number. **b** Thermal conductivity varies as a function of vibrational modes frequency for an amorphous Si, amorphous SiO_2_, and amorphous Si_20_Te_80_. (Reprinted with permission from Ref [99])

Most vibrational modes in amorphous solids are localized as locons, limiting their thermal conductivity [96–100]. Aryana et al. demonstrated that the thermal conductivity of amorphous Si-Te compounds can be tuned by varying the Te composition [99]. As shown in Fig. 17(a) and (b), amorphous Si_20_Te_80_ exhibits the lowest thermal conductivity among amorphous Si-Te compounds. This reduction in thermal conductivity is attributed mainly to changes in the coordination number due to the varying Te content. The study found that in amorphous Si_20_Te_80_, the contribution of propagons and diffusions to the thermal conductivity is negligible compared to amorphous Si and SiO_2_, with locons accounting for approximately 42% of the total thermal conductivity.

Propagons and diffusons predominantly influence the thermal conductivity of amorphous carbon due to the presence of sp^3^ bonds within the material [101, 102]. The ratio of sp^2^ to sp^3^ hybridized bonds, along with the mass density, critically determines the thermal conductivity of amorphous carbon. This hybridization and mass density interplay make amorphous carbon a unique case where thermal conductivity can be engineered by controlling the bond composition and atomic arrangement.

It has been widely accepted that disordered amorphous thin films exhibit lower thermal conductivity than their bulk crystalline counterparts. This is due to the structural disorder, which disrupts phonon transport. Chiritescu et al. demonstrated that disordered WSe_2_ thin films exhibit thermal conductivity values 30 times smaller than their bulk crystalline counterparts [103].

In addition, the study also found that amorphous WSe_2_ films have non-monotonic thermal conductivity changes by thickness. This non-monotonic trend is inconsistent with the general thickness-dependency of 2D materials on the thermal conductivities, where the thermal conductivity decreases with the thickness of 2D materials [104, 105]. In the case of 2D materials, as the thickness decreases, the mean free path of heat carriers (especially diffusons) is significantly shortened due to increased interface scattering, which reduces thermal conductivity. This discrepancy in the amorphous WSe_2_ films is explained by the effective mean free path of diffusions, modulating the interface scattering. In amorphous superlattices with nanometer-scale periodicity, such modulated interface scattering can affect the non-monotonic thermal conductivity changes by thickness below the amorphous diffusive limit, around 1 W/m∙K [106, 107].

**Fig. 18:**
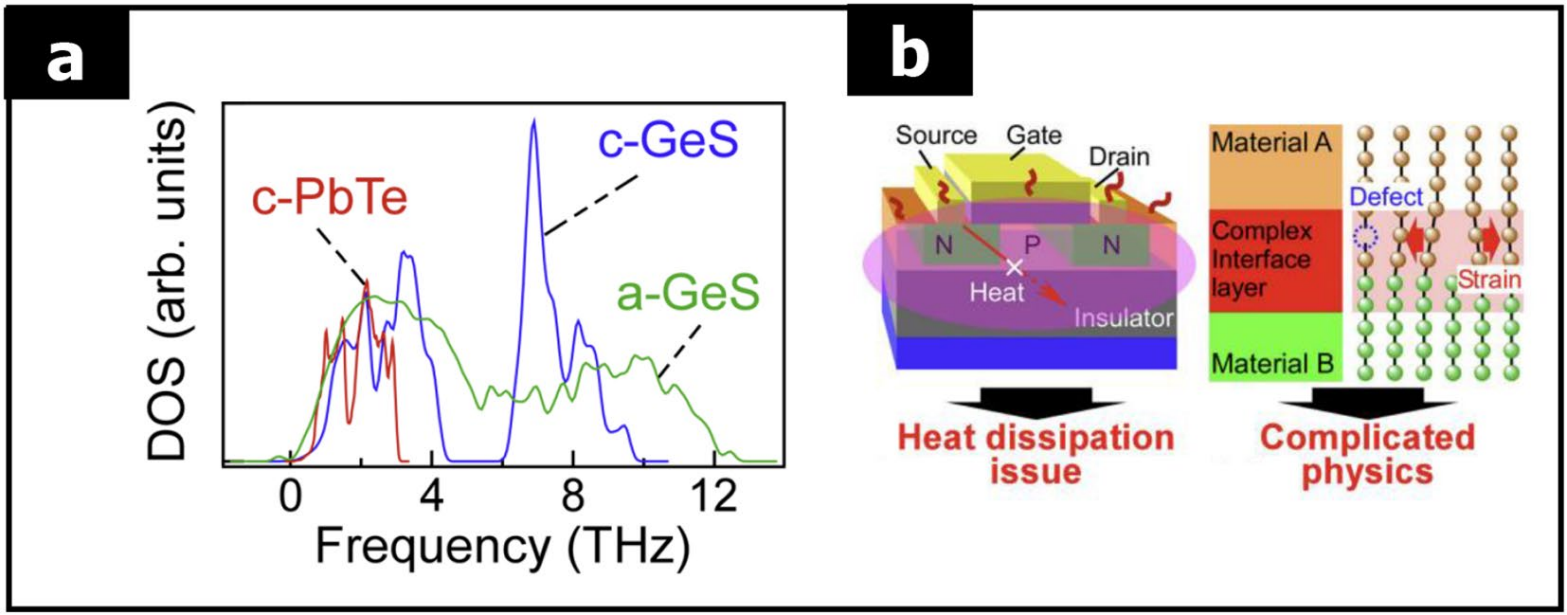
Heat dissipation in a large-scale integrated device. **a** Phonon density of states of crystalline PbTe, crystalline GeS, and amorphous GeS obtained by lattice dynamics. **b** Phonon transport at the interface between materials. (Reprinted with permission from Ref [108])

Another intriguing thermal conductivity aspect arises in heterostructures, particularly for phonon energy matching. In a study comparing conventional phonons in crystalline PbTe and GeS, it was found that the amorphous GeS at the interface between crystalline PbTe and GeS exhibited a notably well-distributed phonon spectrum across a wide range of frequencies (see Fig. 18(a)). This spectrum encompasses all components relevant to thermal conductivity, including propagons, diffusons, and locons [108].

The frequency range of the propagons in amorphous GeS overlaps with that of the crystalline PbTe. This overlap results in lower thermal resistance at the interface and facilitates high phonon transmission through ballistic (energy-matched) thermal transport (see Fig. 18(b)). This case highlights how the structural characteristics of amorphous materials can be leveraged in heterostructures to optimize thermal transport properties, leading to improved performance in applications such as thermoelectric devices and thermal management systems.

## Conclusions

2D amorphous solids have gained increasing attention due to their distinct properties compared to their crystalline counterparts, offering significant potential for advanced electronic devices. While conventional solid-state physics (based on the symmetric lattice) cannot be directly applied to investigate the properties of 2D amorphous solids, we summarized the role of short- and medium-range lattice orders in 2D amorphous solids for their electronic and mechanical properties and phase transition between their amorphous states and crystalline counterparts.

Most deposition techniques, including PVD, CVD, and other modified methods, have been employed to synthesize 2D amorphous solids. However, the challenge lies in optimizing these structures for specific applications, often requiring controlled thermal processes. We note that fabricating ideal amorphous structures is as difficult and critical as creating defect-free, highly crystalline states, particularly for 2D materials where precision at the atomic scale is required.

This review finally highlights three key applications of 2D amorphous solids in sub-nanometer-scale devices: gate dielectric and electric insulators, semiconducting and conducting channels or electrodes, and elements in thermal management systems. Recent studies have demonstrated that the striking advantages of 2D amorphous solids can overcome limitations found in conventional bulk crystals, which is promising and calls for extensive research on this class of materials.

## Data Availability

Any data related to this review are available from the corresponding author on request.
